# Memory Modulation *Via* Non-invasive Brain Stimulation: Status, Perspectives, and Ethical Issues

**DOI:** 10.3389/fnhum.2022.826862

**Published:** 2022-03-04

**Authors:** Mirko Farina, Andrea Lavazza

**Affiliations:** ^1^Faculty of Humanities and Social Sciences, Innopolis University, Innopolis, Russia; ^2^Centro Universitario Internazionale, Arezzo, Italy

**Keywords:** TMS, enhancement, neuroethics, bio-liberals, bio-conservatives

## Abstract

While research to improve memory or counter decay caused by neurodegenerative diseases has a fairly long history, scientific attempts to erase memories are very recent. The use of non-invasive brain stimulation for memory modulation represents a new and promising application for the treatment of certain disorders [such as Post-Traumatic Stress Disorder (PTSD)]. However, numerous ethical issues are related to memory intervention. In particular, the possibility of using forms of non-invasive brain stimulation requires to distinguish treatment interventions from the enhancement of the healthy. Furthermore, a range of important societal and legal concerns arise when manipulating memories. In this short contribution, we address some of the most significant ethical, social, and legal implications surrounding the application of memory-modulation techniques and offer a series of reflections and considerations, which we hope can be of use to guide -and perhaps regulate- their potential, future implementation in society.

## Introduction

Memory can be defined as the faculty of encoding, storing, and retrieving information (Squire, 2009). In the last decades, memory theorists individuated three main types or categories of memory: (i) sensory, (ii) short-term, and (iii) long-term. Each of these kinds of memory has different attributes and characteristics. For example, sensory memory is not consciously controlled, short-term memory can only hold limited information, and long-term memory can store an indefinite amount of information (Zlotnik and Vansintjan, 2019). Answering the question of how memories can be consolidated and processed in the brain quickly became of paramount importance for memory theorists. To date there are many models that attempt to describe how memories are consolidated in cognition (Craik and Tulving, 1975; Conway, 1997; Schacter, 2012).

Some researchers suggested that memory has developed with features useful to the environment and the social life that have characterized much of our evolutionary history (Nesse and Ellsworth, 2009). Threats were forcefully inscribed in our brains (to help us escape them), as were faces (a key fact for in-group interactions). On the contrary, unimportant aspects were soon forgotten, lightening the cognitive load, and making room for newer, more important memories. Other memory theorists, inspired by Vygotsky’s sociocultural theory of human development (Vygotsky, 1962, 1978), emphasized the fact that certain types of memories (such as autobiographical memories) might be also socio-culturally scaffolded (Fivush and Nelson, 2004; Nelson and Fivush, 2004; Harris et al., 2011). That is, they understood memories as being socially mediated processes in which humans may learn how and what to remember and report when talking about past experiences from proactive interactions and collaborative dialogs with more knowledgeable members of society (Sutton et al., 2010) in richly informative environments (Farina, 2020; Ciancarini et al., 2021; Farina and Lavazza, 2021).

Regardless of the specific nature of human memories, manipulating them has been an ancient aspiration of mankind. For a very long time, humans craved to remember better. Think about the method of loci (Yates, 1966). Today, however, thanks to the rapid progress of digital technologies, to the widespread usage of audio-video recording tools, and to the almost immediate access that each of us has to the Web, this original desire for memory augmentation or enhancement has been partly replaced by another (perhaps stronger) desire; the desire or ability to remove bad or negative memories from our lived past.

“Forgetfulness is a form of freedom,” wrote Khalil Gibran. In this sense, and more on this below, the possibility of at least partially controlling the process that leads to forgetting an event, thus avoiding its most stressful emotional aspects, may represent a very valuable goal, unless one endorses a worldview in which suffering may have an instrumental value -e.g., in order to strengthen one’s character or learn something about the phenomenology of life.

With the advent of modern technologies, the possibility of achieving this goal is within our grasp. To date, memory modulation has been used by clinicians to alleviate the suffering of those who have experienced trauma or developed Post-Traumatic Stress Disorder (PTSD). For example, “someone with PTSD often relives the traumatic event through nightmares and flashbacks, and may experience feelings of isolation, irritability and guilt. They may also have problems sleeping, such as insomnia, and find concentrating difficult. These symptoms are often severe and persistent enough to have a significant impact on the person’s day-to-day life^1^.” This is a disorder that can occur with varying severity but is usually highly disabling. In these cases, preventing the re-enactment, with its load of negative arousal, can also improve (or augment) the patient’s condition (Cahill et al., 1994; Pitman et al., 2002).

This is basically the idea of enhancement *via* diminishment (Earp et al., 2014). If we could manipulate memories in this manner, then we could maximize psychological well-being and achieve better and fuller lives. However, modulating memories could also allow those undergoing this procedure to directly improve their working performance, by removing the emotional burden that certain types of memories can impose on the daily routine of an individual. For example, think about a very shy young researcher who does poorly in his first talks and job interviews (cf. Glannon, 2019, 132). The re-enactment of these initial experiences may risk to permanently limit her potential. If, however, the researcher could reduce the strength of her past memories (and more on the potential problems associated with this intervention in section “Ethical and Societal Implications of Memory Modulation” below), the intervention would amount to a kind of enhancement.

In this manuscript, we wish to explore the feasibility as well as the implications underlying the idea of memory modulation. In section “Memory Modulation”, we present a short summary of empirical advancements in research on memory modulation. We then, section “Ethical and Societal Implications of Memory Modulation”, draw the reader’s attention to the potentially significant ethical and societal effects underlying the application of this technique. Finally, we conclude (section “Conclusion”), by calling for the development of a set of shared criteria to distinguish and better discriminate between mere care and empowerment, and briefly reflect on the legal prospects surrounding future usage of memory modulation.

## Memory Modulation

What 40 years ago may have looked like a mere hope (the prospect of modulating memory at will) is within our grasp today. The turning point of this revolution was the discovery of the biological mechanisms underlying memory reconsolidation. We know that memory is not like a tape recorder: it doesn’t faithfully play back our experiences (Bartlett, 1932). Instead, it changes them imaginatively. In other words, memory is reconstructive in character. Building and expanding on this important discovery, recent experimental evidence has amassed demonstrating that memories -when recalled to awareness- enter a phase of lability of the mnestic trace, which is followed by a molecular process called reconsolidation (Nader et al., 2000a,b). In this phase, memories undergo spontaneous “adjustments” of varying magnitudes, which are functional to the context and the new experiences the subject may enact.

Reconsolidation can also be induced though. Extinction techniques have been successfully tested for erasing fear memories (Quirk et al., 2010). For the successful deployment of such techniques, it is of paramount importance to recall the specific memory to be targeted by rapid evocation. This sets in motion the process of reconsolidation, opening a window of lability in the memory itself. Subsequently, in a time span that does not seem to exceed a few hours, the extinction procedure in the proper sense is practiced. This consists in repeating the presentation of the conditioned stimulus no longer followed by the unconditioned stimulus. Repetition in succession weakens the stimulus-response connection leading to the extinction of the associative memory (Schiller et al., 2010; Xue et al., 2012).

Based on this knowledge, attempts have been made to directly interfere with reconsolidation. After the reactivation of one’s memory, one can intervene chemically to weaken or erase it. In animals, this has been done with anisomycin, an inhibitor of protein synthesis that underlies the molecular process of memory reconsolidation (Wang et al., 2005). This procedure seems to be quite effective; however, some limitations also apply. For example, anisomycin is toxic for humans. In addition, it still seems too great the risk involved in performing an action that -despite being specifically targeted to a single memory trace- may affect, in an uncontrolled way, a series of different memories.

Here an argument against the feasibility of deleting individual memories can be introduced, precisely because memories are usually closely interconnected by content -as shown by their resurfacing in clusters and/or in a concatenated way- in terms (for instance) of similarity or spatiotemporal contiguity. However, it should also be noted that in the medial temporal lobe, researchers (e.g., Quiroga, 2012) identified single neurons called “concept cells,” capable of responding selectively and abstractly to specific people and objects (such as Jennifer Aniston or the Tower of Pisa).

Considering the above-mentioned limitations underlying the usage of anisomycin in humans, researchers started using another molecule (propranolol) to achieve their goal. Propranolol is a beta-blocker that has been tested for some years to counter traumatic memories and the onset of PTSD (Brunet et al., 2018). Propranolol is a molecule that has been shown to have some effect in mitigating the emotional burden of memories when taken a few hours after the negative event (Kroes et al., 2016). For the sake of completeness, it must be noted here, that post-learning β-adrenergic blockade has been shown to impair memories in mice only when training was conducted in high arousing conditions (Conversi et al., 2014). The semantic memory of the event is not altered, and the physiological activation associated with the emotions appears to be strongly reduced (Kindt et al., 2009).

Recently, it has also been shown that repetitive transcranial magnetic stimulation (rTMS) performed on the dorsolateral prefrontal cortex can disrupt the reconsolidation of fear memory and prevent its return. Researchers found out that this type of intervention is state-dependent. More precisely, they “stimulated the dorsolateral prefrontal cortex (dlPFC) 10 min after a reminder cue that reactivated a fear memory acquired 1 day before. At testing, 24 h after rTMS, participants exhibited decreased physiological expression of fear, as shown by their skin conductance response” (Borgomaneri et al., 2020, 3672). The effect was not observed when the reconsolidation process was not triggered or when subjects were tested for the expression of fear immediately after stimulation. On these grounds, the researchers claimed that such interventions are effective only if they occur within the reconsolidation time window. Furthermore, it seems that dlPFC has a relevant causal role in fear-memory reconsolidation processes. Moreover, it was noted that “dlPFC-rTMS prevented subsequent return of fear after extinction training” (Borgomaneri et al., 2020, 3672).

While other studies will have to replicate these findings as well as better understand what kind of memories are amenable to modification and to what extent, non-invasive brain stimulation could quickly become an easily administrable tool for memory modulation. For instance, it could be safer and more controllable than drugs. However, since private medical practices are already offering non-clinical treatments with TMS to improve the well-being of healthy subjects, ethical and societal implications surrounding the widespread usage of such techniques should be addressed with some urgency.

## Ethical and Societal Implications of Memory Modulation

In truth, the process of memory modulation *via* non-invasive brain stimulation techniques has already received some attention in the relevant bioethical literature (for an overview see, Lavazza, 2019). One can say that there are two paradigms rivaling each other. One paradigm is known as the “bio-liberal” approach (Buchanan, 2011); the other, as the “bio-conservative” view (Lavazza and Inglese, 2013; Glannon, 2019).

The bio-liberal approach defends the idea of individual autonomy as grounded on the “harm principle” proposed by Mill (1859), according to which: “the only purpose for which power can be rightfully exercised over any member of a civilized community, against his will, is to prevent harm to others”. Proponents of this paradigm assert that the subject is free about the decisions to be made for herself, her conduct, and her life. Autonomy -on such a view- is thus generally understood as the ability to be one’s own person, to live one’s existence according to reasons and motives that one assumes as one’s own and not as the product of manipulative or deforming external forces. Those who are autonomous can decide for themselves without interference from others or without personal limitations; they can act according to their own plan, undertaken without constraint. This bio-liberal approach thus allows for the implementation and adoption of potentially any therapy, so long as it can bring a perceived net benefit to the individual (Buchanan, 2011).

The bio-conservative view values instead the authenticity of the subject. The authenticity of the subject consists of all those distinctive traits that ensure personal continuity or identity, and to which one should remain faithful. Authenticity (understood as second-order identification of one’s desires: Frankfurt, 1977) represents the consistency of the individual’s choices with her identity (at a given time), or at least with those components of her identity that are relevant to the choice/decision being made. Because memory modulation is likely to change authenticity hence personal identity, the bio-conservative approach cannot approve of such a practice (Kass, 2003; Sandel, 2009).

One might wonder though about why authenticity is or should be valued. If one makes choices that are not attuned with one’s identity, what follows is a forced change of self: a form of betrayal. Respect for authenticity, for bio-conservatives, is thus a form of honesty and fidelity to the “true self.” As far as social aspects are concerned, giving up authenticity may determine a leap into unreality: inauthenticity often implies non-adherence to facts (wanting to be someone else). Finally, coherence is often considered as a value on its own, or -at least- in adaptive terms as a function that linked with social reliability ensures many benefits to the individual who displays it.

So far in the literature, interventions on memory that have a specific enhancing character (*via* diminishment) have not been widely discussed; or the examples discussed indicate the difficulties in evaluating new potential scenarios and devising a common, unitary framework for guiding and explaining them.

Erler (2011) describes a situation in which a girl, Liz, who has long been bullied at school has now become (also because of that bullying) a right activist. However, she is still unable to reconcile with her fellows. Liz could resort to a memory-modulation intervention, which may lead her to be less obsessed by her past and more socially integrated. With the intervention her commitment to fighting bullies will fade. Fighting bullies, though, seems to be a constitutive part of her personality and what makes for her positive contribution to society. Should the intervention take place then?

In a similar vein, Lavazza (2015) imagines that in a masculinist society, subjugated women may resort to pharmacological modulation of memories to endure their situation. In this way, the potential for resistance and struggle against societal abuse would be diminished by an unintended compounding effect, due to which women will not be able to emancipate themselves. More recently, Zawadzki and Adamczyk (2021) point out that in interventions (based on optogenetics) performed for the removal of painful memories, there may be loss of opportunities for those involved to positively reframe their existential events (due to lack of awareness).

If interventions aimed at alleviating the suffering of patients affected by serious forms of PTSD seem to be acceptable by both the ethical paradigms we have briefly reviewed; those interventions concerning modulation of memories in healthy individuals discussed in the two paragraphs above seem to be far more problematic as they constitute ground that is still somehow unexplored. Specifically, there seems to be a difficulty in drawing a clear line between what is treatment and what is enhancement. This is a problem that -to date- in bioethics has not found a universally accepted solution (Schwartz, 2005).

Consider again the example we discussed at the beginning of this manuscript, involving the shy researcher. Early failures caused by an innate feature of her character were dysfunctional to her social and professional advancement and could be classified as pathological elements that should be treated. However, as pointed out by Glannon (2011), if through an act of memory remodulation, the shy researcher could erase the memory or at least the emotional burden of his failures, she could enter a dimension of detachment from reality. Others would still see and know about her history of failures, but she wouldn’t be aware of them. This fact seems to imply that subtractive memory remodulation (or enhancement *via* diminishment) may have risky side effects.

Consider the following case (concerning individuals determined to engage in criminal conduct) as a further illustration of this claim. In many cases these individuals are potentially restrained from committing a wrongful act by a multiplicity of factors, including: (i) deterrence (the fear of punishment), (ii) potential remorse, and/or (iii) guilt. Now, imagine a situation where all such individuals could reliably access tools or techniques (such as non-invasive brain stimulation) through which they could erase those memories of fear and distress. The crime rate would surely increase (just as there is a black market for weapons, there could be one for TMS; and just as many very good chemists get into the illegal drug trade, so experts in neurotechnology could be willing to implement any kind of TMS application for profits). Even armed forces might want to disrupt memories or dampen their emotional component, so that their soldiers would not be conditioned by feelings of horror or pity when deployed in combat operations (Adamczyk and Zawadzki, 2020).

Yet, one could also imagine an example in which the memory remodulation would violate the authenticity of the individual but would still be respectful of deontological aspects (such as autonomy) and even contribute to accomplish good societal results. This is the case of a person who may seek to mitigate particular memories of her childhood, that could limit her in acquiring an entrepreneurial capacity, which would subsequently allow her to become a successful businesswoman capable of helping those living in less fortune conditions (Lavazza, 2021).

It is unlikely that memory modulation will become readily available in private medical practices and that one could rewrite her biography at will; and even if such possibility will arise in the future it won’t be likely allowed without a clear therapeutic goal. However, the nuanced cases (such as those discussed in this short contribution) are likely to be those that will need more attention and closer ethical and legal scrutiny. Movies such as [*Eternal Sunshine of the Spotless Mind* (2004) and *8 New Dates* (2015]) anticipated scenarios in which one would be able to get rid of past loves. Disappointments of love or abandonment can certainly become traumatic and cause pathological consequences. Trying to modulate memories of many of our experiences could nevertheless prove to be deeply dysfunctional. One effect might be not only a loss of a sense of reality, but also the inability to come to terms with failures and learn from negative experiences.

## Conclusion

We believe we should not get swept up in the hype about non-invasive brain stimulation techniques/technologies, as far as memory interventions are concerned. At the same time, it is important to encourage research in the field, as research will keep trying to develop effective tools to treat severe forms of PTSD, and it is not excluded that new forms of memory modulation will be available in the near future. For this reason, it seems reasonable to suggest that we begin to consider what may be the implications of forms of enhancement achieved with a reduction in memory (either with a complete deletion or - if it will be possible- with a mitigation of their emotional and negative load).

In this context, it also seems of paramount importance that researchers start formulating a set of shared criteria to distinguish and discriminate between care and empowerment. In addition, experts should carefully review and debate recommendations and possible adjustments for those who want to undergo memory modulation interventions for enhancement. It is also time to think about how to sensitize scientists, physicians, as well as manufacturers of non-invasive brain stimulation devices to the ethical and societal implications of certain kinds of memory modulation. The production of guidelines or even of a moratorium, in certain areas, could be a desired outcome in this respect.

In wrapping up this short contribution we would also like to briefly reflect on the legal implications underlying memory modulation. These implications should not be so easily dismissed or underestimated, as they could significantly affect (either directly or indirectly; *via* modification of personal identity) the commitments and obligations that individuals might have assumed during their lives. For example, should an individual who genuinely forgot that she had committed a crime be charged and convicted? Could an individual who did not remember being married be forced to keep all her marital obligations?

Proposing a bio-liberal answer to these important questions, based on a strict adherence to the harm principle above-mentioned might turn out to be very problematic. It seems to us that the tenet according to which the individual is free to the extent that it does not cause harm to others implies that the freedom of modifying memories defining the individual’s identity should necessarily be somehow limited; otherwise, any legal system would risk collapsing (Marshall, 2014). A society based on a legal system requires a quite stable personal identity; else (as per examples discussed above), a person found guilty of a crime could request the cancelation of her key memories concerning personal identity and then, claiming to have become “another” individual, also request the annulment of the sentence.^2^ Similarly, a disloyal husband may become a womanizer and cease to support his family, without having to face the legal and financial consequences of his decision. However, it should also be noted that such a radical change of personal identity cannot be brought about with memory-erasing, except with the loss of one’s identity as such.

It thus seems sensible, for these reasons and (probably) for more, to suggest the need to carefully assess and evaluate the potential future implications of non-invasive brain stimulation for memory modulation interventions right now; with sobriety and a sense of equilibrium, which demands neither hyping results nor invoking principled prohibitions that appear to be vastly overstated given current progress (or lack thereof) in the field.

## Author Contributions

Both authors contributed equally to the writing of this manuscript and approved the submitted version.

## Conflict of Interest

The authors declare that the research was conducted in the absence of any commercial or financial relationships that could be construed as a potential conflict of interest.

## Publisher’s Note

All claims expressed in this article are solely those of the authors and do not necessarily represent those of their affiliated organizations, or those of the publisher, the editors and the reviewers. Any product that may be evaluated in this article, or claim that may be made by its manufacturer, is not guaranteed or endorsed by the publisher.

## Footnotes

1 https://www.nhs.uk/mental-health/conditions/post-traumatic-stress-disorder-ptsd/overview/ Last accessed December 2021.

2 ​Thanks to the reviewer for pressing us on this point.
